# Machine Learning for Detection of Muscular Activity from Surface EMG Signals

**DOI:** 10.3390/s22093393

**Published:** 2022-04-28

**Authors:** Francesco Di Nardo, Antonio Nocera, Alessandro Cucchiarelli, Sandro Fioretti, Christian Morbidoni

**Affiliations:** 1 Department of Information Engineering, Università Politecnica delle Marche, Via Brecce Bianche, 60131 Ancona, Italy; s1102518@studenti.univpm.it (A.N.); a.cucchiarelli@staff.univpm.it (A.C.); s.fioretti@staff.univpm.it (S.F.); 2 Department of Management and Business Administration, University of Chieti-Pescara, 65127 Pescara, Italy; christian.morbidoni@unich.it

**Keywords:** onset detection, muscle activation, machine learning, neural networks, surface EMG

## Abstract

Background: Muscular-activity timing is useful information that is extractable from surface EMG signals (sEMG). However, a reference method is not available yet. The aim of this study is to investigate the reliability of a novel machine-learning-based approach (DEMANN) in detecting the onset/offset timing of muscle activation from sEMG signals. Methods: A dataset of 2880 simulated sEMG signals, stratified for signal-to-noise ratio (SNR) and time support, was generated to train a hidden single-layer fully-connected neural network. DEMANN’s performance was evaluated on simulated sEMG signals and two different datasets of real sEMG signals. DEMANN was validated against different reference algorithms, including the acknowledged double-threshold statistical algorithm (DT). Results: DEMANN provided a reliable prediction of muscle onset/offset in simulated and real sEMG signals, being minimally affected by SNR variability. When directly compared with state-of-the-art algorithms, DEMANN introduced relevant improvements in prediction performances. Conclusions: These outcomes support DEMANN’s reliability in assessing onset/offset events in different motor tasks and the condition of signal quality (different SNR), improving reference-algorithm performances. Unlike other works, DEMANN’s adopts a machine learning approach where a neural network is trained by only simulated sEMG signals, avoiding the possible complications and costs associated with a typical experimental procedure, making this approach suitable to clinical practice.

## 1. Introduction

Assessing muscle-recruitment timing is relevant in different fields, including clinical gait analysis and electromyography-driven assistive devices [1,2]. Traditionally, onset/offset events are detected by visual inspection of surface electromyographic (sEMG) signals by trained experts [3]. However, visual inspection may be time-consuming, not completely reproducible/repeatable, and not suitable for large datasets [4]. A further classical approach is represented by threshold-based automatic methods [5]. Among these, the double-threshold statistical algorithm (DT) is a robust approach, and nowadays it is still widely adopted for clinical and research purposes [6,7]. Further approaches are typically developed based on time-frequency analysis [8,9,10,11] and signal filtering by a Teager-Kaiser energy operator (TKEO) [12]. As reported [4,13], performances of the above-mentioned approaches could be significantly affected by the relative amount of background noise compared to the magnitude of the actual sEMG signal, i.e., low values of the signal-to-noise ratio (SNR). A further issue to consider is that the majority of these approaches do not take into account those conditions where SNR is not constant throughout the signal acquisition, such as during prolonged tasks (walking, running, cycling). Intra-signal variability of SNR during sEMG recording could be ascribed mainly to the change of noise power, due to the alteration of electrode–skin contact characteristics or to the changes in the ground reference level [7]. This could strongly affect the onset-offset event detection in those portions of the sEMG signal where SNR deteriorates.

Machine/deep learning has proven to be effective in interpreting sEMG signals for different purposes [14], such as to classify gestures [15], to detect muscle fatigue [16], and to investigate human–machine interaction [17]. Different models were adopted: convolutional and recurrent neural networks for muscle force estimation [18], unsupervised competitive learning for assessing muscle recruitment during pregnancy [19], and multi-layer perceptron to classify neuromuscular disorders [20,21]. Support vector machines were largely applied to the sEMG signal for classification purposes [22,23] and for the detection of physiological patterns and parameters [24,25]. Attempts were made even for characterizing the walking task, with particular focus on classifying gait phases and assessing gait [26,27,28,29].

In spite of the presence of a large literature on the machine-learning based interpretation of sEMG signals, this approach is scarcely adopted to face the challenge of assessing the timing of muscle activation. The problem to solve is essentially an sEMG-based prediction of a transition between the period when the muscle is silent and the period when the muscle is active, i.e., to discriminate between actual sEMG activity and noise. Given that, the possibility of adopting a machine learning approach that learns to interpret the shape of the sEMG signals for assessing muscle-activation onset and offset seems to be a feasible solution. A very recent study proposed by Ghisleri et al. adopted long short-term memory (LSTM) recurrent neural networks (RNN) for detecting muscle activity [30]. Very encouraging outcomes were achieved in this study by using a very diversified dataset of sEMG signals to train the network, including simulated signals, signals from able-bodied subjects, and signals from patients affected by neurological or orthopedic pathologies. To run this approach, a large dataset of real sEMG signals from many different subjects is needed. However, recruiting an adequate number of subjects to build the dataset could be a challenging task. This is particularly true if patients affected by different pathologies are included, as in this case. Thus, an alternative way that considers a less demanding approach to neural-network training could be valuable. A first preliminary (and at the moment the only) attempt to provide a different approach to the training phase was proposed, based on the idea of including only simulated sEMG signals in the training procedure [31]. This study used the wavelet spectrogram of sEMG signals as the input to the network. Model performances were provided only in terms of absolute latency of onset-timing detection. Validation performed against two literature methods [32] showed promising results in terms of latency, encouraging research to continue along this path. However, the prediction of the offset event is not provided, and the model performances are tested only in a single subject, questioning the clinical impact of this approach and the reliability of the validation procedure.

The goal of the present study was to investigate the suitability of a novel machine-learning-based approach in assessing the onset-offset timing of muscle activation, i.e., the Detector of Muscular Activity by Neural Networks (DEMANN). Specifically, the present approach aimed to predict both onset and offset timing using only simulated sEMG signals with a large range of SNR values for neural network training in order to explore a large range of SNRs without deterioration, which is often encountered in clinical environments. This aspect, together with the simple architecture of the neural network (based on a multi layer perceptron), should help to provide fast training and prediction, making this approach very suitable for clinical purposes. Thus, the main contributions that the present study would like to provide could be summarized as follows:

- To develop a novel high-performance approach (DEMANN) that contributes to support the use of machine learning for muscle activity detection;
- To highlight the advantages of the proposed machine-learning approach, such as the possibility of real-time applications, achieved without loss of accuracy and with respect to existing, non-machine-learning-based systems;
- To limit the deterioration of event assessment associated with low SNRs and the large inter-signal variability of SNR, typical of clinical environments, by training the model with simulated sEMG signals with a large range of SNR values;
- To reduce the complexity of the experimental protocol associated with model training, since no signal acquisition is needed to provide real time activation predictions.

## 2. Materials and Methods

The robustness of DEMANN was evaluated by a test bench of simulated sEMG signals and two datasets of real sEMG signals. Simulated and real sEMG signals underwent the same procedure described in the following sections. DEMANN was validated by a direct comparison with reference approaches on both simulated and real data.

### 2.1. Simulated sEMG Signals

A simulation study, using a test bench of signals, was carried out for assessing the performance of the DEMANN approach in predicting onset and offset events of muscular activity. sEMG signals acquired during cyclic movements could be modeled as the superimposition of the actual signal produced by muscle contraction and the background noise [33]. In this study, a Gaussian process with zero mean and variance $\sigma_{noise}^{2}$ was adopted to model the sEMG-signal where the muscle was silent and only background noise was acknowledged. To simulate the sEMG-signal portion where the muscle is recruited, the background uncorrelated noise was added to a band-limited stochastic process with zero-mean Gaussian distribution of amplitude and a fixed power level [6]. This distribution was achieved by band-pass filtering (80–120 Hz) a Gaussian series of uncorrelated samples, according to [6]. This Gaussian distribution was truncated to simulate the sEMG activity due to muscle activation. Each simulated sEMG signal was generated with a sampling frequency fs = 2000 Hz, a time window = 1 s, and a variable value of the Gaussian-distribution median, µ, ranging from 0 to 1. Different simulated sEMG signals were created varying the standard deviation, σ, and the time support, 2 × α × σ, of the Gaussian distribution, in order to simulate the physiological variability associated with the recruitment of different muscles. The variation of σ was achieved according to the desired value of SNR, where:

(1) $$\mathrm{SNR}=10*\log\frac{\left(\sigma_{signal}^{2}\right)}{\left(\sigma_{noise}^{2}\right)}$$

Simulated sEMG signals were generated from all the different combinations of the values adopted for σ (50, 100, and 150 ms), for α (1, 1.5, 2, and 2.4), and SNR values from 1 dB to 30 dB, with step = 1. In [30], Ghisleri et al. trained LSTM recurrent neural networks by means of simulated sEMG signals, with SNR ranging from 3 dB to 30 dB. In the present paper, this SNR range was slightly expanded to consider even worse conditions.

### 2.2. Real sEMG Signals

Two different datasets of real sEMG signals were considered. The first dataset is available in [3] (https://github.com/TenanATC/EMG, accessed on 23 April 2021), including the ground truth. The experimental protocol consisted of acquiring sEMG signals from 18 participants performing knee extension and elbow flexion. Knee extension was performed in subjects seated in a stationary chair, with a mass (2.3 kg) applied to the right ankle. Elbow flexion was performed with a mass (2.3 kg) applied to the right wrist. sEMG probes were applied over vastus lateralis (VL) for monitoring knee extension and over biceps brachii (BB) for elbow flexion. A total of 103 sEMG signals were acquired with 0 dB < SNR < 13 dB. Three experts visually analyzed the signals and noted down the activation onsets in a randomized and double-blind fashion. Every trial was inspected twice by each expert. The average over the six onset values was the ground truth for the experiments in [3] and it was adopted also here. Further details can be found in [3].

The second dataset consisted of foot–floor contact and the sEMG data collected during 30 healthy adults walking, retrospectively taken from the database built at the Movement Analysis Lab, Università Politecnica delle Marche, Ancona, Italy and used for previous studies [28,29]. Data are freely available, consulting the public repository of medical research data PhysioNet [29,34,35]. Overweight and obese people (body mass index, BMI > 25) and subjects affected by any pathological condition, joint pain, or undergone orthopedic surgery were not considered. Gait data were captured (sampling rate: 2 kHz; resolution: 12 bit) by the multichannel recording system Step32 (Medical Technology, Torino, Italy). sEMG signals were acquired in each leg by single differential probes placed over gastrocnemius lateralis (GL), tibialis anterior (TA), and vastus lateralis (VL). SNR values ranged between 3 dB and 30 dB. SENIAM guidelines for sEMG-sensor positioning were respected [36]. Foot–floor contact signals were measured by three footswitches placed under the heel and the first and the fifth metatarsal heads of the foot. Subjects walked barefoot at a self-selected pace for about 5 min, following an eight-shaped path, which involved natural deceleration, acceleration, and reversing. Further details are reported in [28]. The research was undertaken following the ethical principles of the Helsinki Declaration and was approved by the local ethical committee.

### 2.3. Signal Pre-Processing

Simulated and real sEMG signals were band-pass filtered (2nd-order Butterworth filter, cut-off frequency 10–500 Hz). Then, signals were pre-processed to extract the linear envelope (LE), the root mean square (RMS), and the wavelet scalogram, which were concomitantly used as input to the neural network. LE was extracted by low-pass filtering of the signal (2nd-order Butterworth filter; cut-off frequency 5 Hz). RMS was extracted by computing the following formula over overlapping sliding 60-sample windows that scan the whole signal:

(2) $$\mathrm{RMS}=\sqrt{\frac{1}{T}\;\int_{0}^{T}{\left|x\left(t\right)\right|}^{2}\;dt}$$

Continuous wavelet transform (CWT) was used for providing energy localization in the time-frequency domain of sEMG signals in terms of CWT scalogram function, *P_sEMG_*, defined as the square of the absolute value of CWT coefficients, *W_sEMG_*:

(3) $$P_{sEMG\;}\left(a,b\right)=|W_{sEMG\;}\left(a,b\right){|}^{2}$$

Wavelet transform was implemented by adopting Morse of order 4 with 6 levels of decomposition as mother wavelet.

### 2.4. Data Preparation

**Figure 1:**
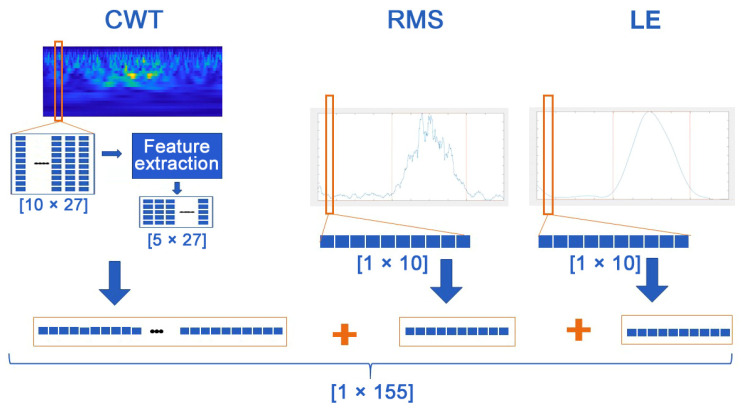
Realization of sEMG vectors used as input to DEMANN model.

To adopt the most suitable input to the neural network, preliminary experiments were performed, evaluating four different alternatives: LE, RMS, CWT scalogram, and their concatenation (LE + RMS + CWT). The concatenation consisted of a min–max normalization of the outputs of the different processing procedures, thus mapping the values in a [0, 1] range, and a concatenation of outputs of the different processing procedures (Figure 1). These choices were motivated by the related literature, where LE and RMS of the sEMG proved to be suitable signals to train the neural network for gait analysis [27,28,29], even if the prediction tasks were different from the one addressed here. Outputs of time-frequency analysis (spectrograms, scalograms) were also features often used in sEMG analysis, as for example in [31] to predict muscle activations. Before training the classifier, the concatenated vector was segmented in overlapping sliding windows of 10 samples, where each window was shifted of one sample with respect to the previous window. Each window was used to label that single sample, according to the value of the related ground truth in the window. The single sample was labeled as 1 (muscle activity) or 0 (no muscle activity), according to the most frequent ground truth value identified in the window. The size of the processed windows, the simple neural network architecture, and the use of sliding windows provided a very low latency of 3–4 milliseconds, which could be suitable for real-time applications.

### 2.5. Training the Classifier

The classifier was a hidden single-layer (32 units) fully-connected neural network. A Rectified Linear Unit (ReLU) activation function was used, and a sigmoid function was adopted to map the network output to a 0–1 interval. The binary output was achieved by using a standard threshold of 0.5. The model was trained with a learning rate of 0.001, a batch size of 512 for 40 epochs using the standard stochastic gradient descent (SGD) optimization algorithm, and by adding a L2 regularization penalty set to 0.0001. The training set was composed of only simulated sEMG signals: 8 signals for each combination of σ (50, 100, and 150 ms) and α (1, 1.5, 2, and 2.4) were chosen, for a total of 96 signals for each SNR. Considering 30 SNR values (from 1 dB to 30 dB, step = 1), a total of 2880 simulated signals were included.

The classifier performances were evaluated on three different testing sets. The first one was composed of only simulated sEMG signals. Eight signals were generated for each combination of σ, α, and SNR. Nine different SNRs were considered, specifically 3, 6, 10, 13, 16, 20, 23, 26, and 30 dB, as suggested in [6]. A total of 864 simulated signals were achieved. No overlapping occurred between the training and testing set, i.e., none of the simulated signals generated to train the model were used during testing. The ground truth of muscle activity was the vector composed of the same number of samples of the simulated sEMG signal, where samples can assume only two values: “0” and “1”. The ground truth was “1” if the truncated Gaussian distribution assumed values > 0, “0” otherwise. The DEMANN performance was provided in terms of precision, recall, F1-score, and mean absolute error (MAE), assessed in true positives as defined in Section 2.6. MAE was the average time distance between the predicted event and the one of the same kind in the ground truth signal. A comparison of the results achieved in the first test set was reported in Table 1, in terms of the mean F1-score (±SD) of classification. The overall best F1-score was achieved by LE + RMS + CWT (Table 1). Thus, this input was adopted to feed the neural network.

The second test set was composed of 103 real sEMG signals proposed in [3]. The performance of the DEMANN approach was provided in terms of prediction accuracy and MAE, assessed in all 103 signals of the dataset.

The third test set included foot–floor contact and sEMG data collected during 30 healthy adults walking, as described in Section 2.2. Sequences of five consecutive gait cycles were selected randomly. Two experts analyzed three different versions of the same signal: raw sEMG signal, rectified band-pass-filtered sEMG signal, and RMS of the sEMG signal. Then, the experts identified onset-offset instants of muscular activity by visual inspection. The mean over the six onset values represented the ground truth for the experiments. A total of 538 events were identified (269 onsets and 269 offsets). The reference chosen for validation was the acknowledged double thresholding algorithm (DT) [5,6]. The performances were reported in terms of precision, recall, and F1-score of the event prediction.

For all the three test sets, model validation and performance were computed in signals never used during the training of the model.

### 2.6. Identification of sEMG Onset-Offset

To achieve the model output, segmented sEMG signals were provided as input to the trained model. Thus, the model output was composed of sequences of 0 (no muscle activity) alternating with sequences of 1 (muscle activity). This signal was chronologically scanned to identify the transitions between the two conditions: the transition from 0 to 1 identified the onset event and the transition from 1 to 0 detected the offset event. This was achieved by the following procedure: a time tolerance *T* of 100 ms was adopted, as suggested in [10]. Then, we acknowledged as true positive each predicted event at time *t_p_* if an event of the same kind occurred in the ground-truth signal at time *t_g_*, such that $\left|t_{g}-t_{p}\right|<T$. Otherwise, the predicted event was acknowledged as false positive. Moreover, a post-processing procedure was performed, consisting of cleaning the signal by discarding those sequences of samples that were too short to be physiologically plausible; it was acknowledged, indeed, that muscle recruitments lasting less than 30 ms had no effect in controlling joint motion [6]. Thus, sequences of 0 (or sequences of 1) shorter than 60 samples were removed.

### 2.7. Statistics

The Shapiro-Wilk test was adopted to appraise the normality of data distribution. A two-tailed, non-paired Student’s *t*-test was applied to verify the significance of difference between the normally-distributed samples. The Mann-Whitney test was applied to verify the significance of difference between the non-normally-distributed samples. Statistical significance was established at 5%.

## 3. Results

### 3.1. Simulated sEMG Signals

The mean classification accuracy computed in the testing set stratified for different SNR is shown in Table 2. The accuracy on the simulated test set increased with increasing SNR from 3 dB (accuracy = 95.3%) to 23 dB (accuracy = 99.2%), and then it remained practically unaltered. Likewise, SD decreased with increasing SNR (from 4.8 to 0.7%).

Table 3 reports the mean classification performances in the testing set computed separately in the portions of sEMG signals where muscle activity was acknowledged (activity area) and where it was not (silent area). The effect of SNR on the classification performances was preserved.

While in the present study, a shallow neural network was used as a classifier, the DEMANN approach can be flexibly modified to embed a different machine-learning model. Support vector machines (SVM) are identified in literature as suitable modeling tools [22,23,24,25]. Thus, a direct comparison was performed, with results achieving replacing the neural network with a linear kernel SVM classifier on the same dataset of simulated sEMG signals. The SVM model was trained with the Stocastic Gradient Descent optimizer on a Hinge loss function and by applying a L2 regularization with coefficient 0.0001. The results of this comparison are shown in the following Table 4.

A significantly lower mean MAE (*p* < 0.05) was provided by the DEMANN approach for both onset and offset timing. No significant differences were detected in precision, recall, or F1-score between the performances of the two models.

**Figure 2:**
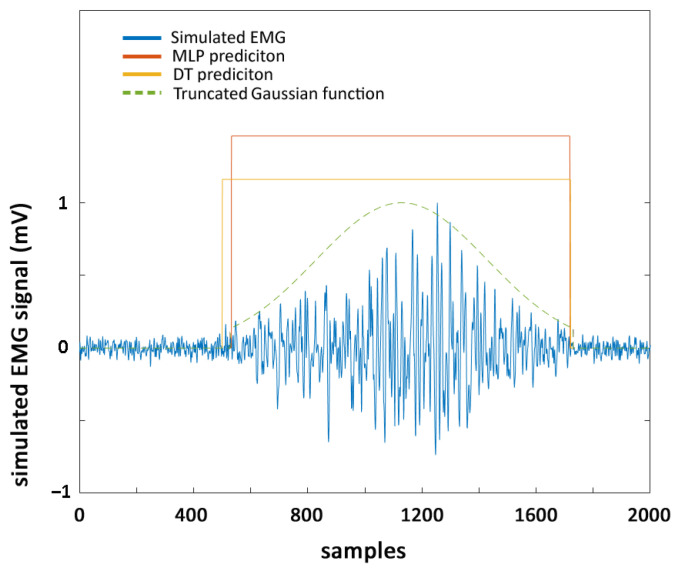
Example of simulated sEMG signal (blue line). The truncated Gaussian function used to model the simulated signal (green dashed line), predictions by DEMANN (red rectangle), and DT (yellow rectangle) of onset and offset events are superimposed.

Figure 2 reports an example of simulated sEMG signal, where onset and offset events predicted by DEMANN and DT approaches (rectangular lines) are highlighted and compared with the ground truth, i.e., the truncated Gaussian function used to model the simulated signal.

The average performances of the onset-offset prediction over the simulated-signal dataset provided by the DEMANN and DT approaches are reported in Table 5.

The variability of MAE in the function of α, σ, and SNR is quantified in Table 6. A color-level coded representation was adopted to allow a visual interpretation of results.

**Figure 3:**
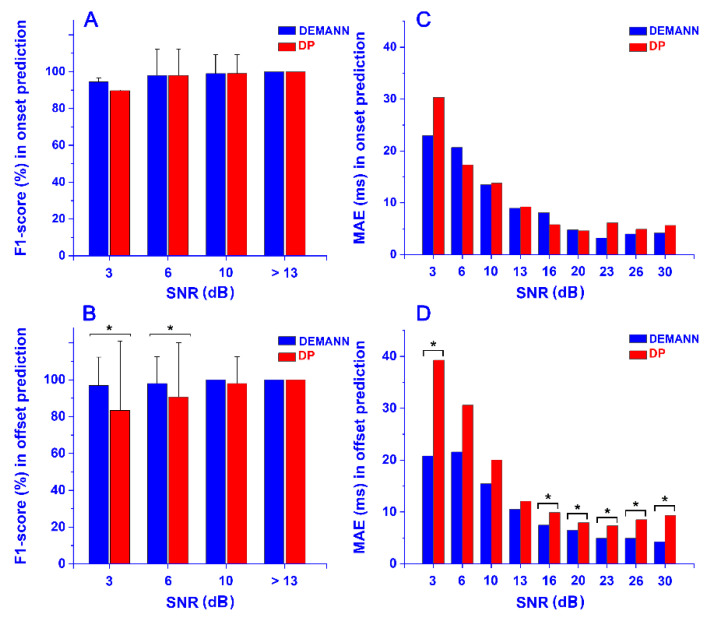
Mean F1-score computed in onset (panel **A**) and offset (panel **B**) prediction and mean MAE computed in onset (panel **C**) and offset (panel **D**) prediction for each SNR value by DEMANN (blue bars) vs. DT algorithm (red bars). * indicates statistically significant difference.

The direct comparison of performances achieved by DEMANN and DT is depicted in Figure 3, stratified for different SNR. An improvement of the F1-score of offset prediction was introduced by DEMANN for signals with SNR ≤ 6 dB (*p* < 0.05, Figure 3B). No significant differences were detected for SNR > 6 dB. The F1-score was comparable for onset prediction in the whole SNR range (*p* > 0.05, Figure 3A). Lower MAEs in onset-offset prediction were provided by DEMANN. Details of statistical significance are reported in Figure 3C,D.

### 3.2. Real sEMG signals

A first validation was performed on the sEMG dataset available in [3]. In [13], four onset-detection algorithms and two filtering approaches were tested on this dataset characterized by SNR ≤ 8 dB. The same 52 sEMG signals were considered here (first four lines, Table 7).

As in [13], the 52-signal dataset was split according to four ranges of increasing SNR values (step = 2 dB) to facilitate the comparison of results. The absolute error of the onset prediction provided by DEMANN is reported in Table 7, in terms of mean, standard deviation (SD), median, 25-percentile, and 75-percentile. Validation was performed against the four algorithms tested in [13]: the double-threshold statistical algorithm (DT) [6]; the wavelet-based approach (WLT) [9]; the method grounded on CUSUM logic [37]; and the technique based on profile-likelihood maximization, employing discrete Fibonacci search (PROLIFIC) [38]. DEMANN provided the lowest values of absolute error for all the metrics (Table 8), except for SD (best value = 114.8 ms; DEMANN-value =120.3 ms). Similar consideration could be performed for signals with 6 < SNR < 8 dB. For lower SNR (<6 dB), DEMANN provided performances comparable to the other algorithms (Table 8). The results of signals with 8 < SNR < 12 are also reported in Table 7. Precision, recall, and F1-score were dependent on the choice of the tolerance used to identify true positives. In this case, all the events were detected within the tolerance range, leading to a precision, recall, and F1-score of 100% for DEMANN and for all the algorithms chosen for validation.

**Figure 4:**
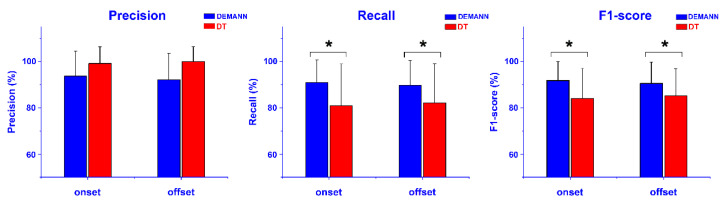
Mean (±SD) precision, recall, and F1-score computed in the onset and offset prediction by the DEMANN approach (blue bars) vs. the DT algorithm (red bars) achieved in real sEMG data during able-bodied walking. * indicates statistically significant difference.

A second validation was performed on the sEMG dataset acquired during walking (Section 2.2), with a direct comparison to the DT algorithm. Outcomes are reported in Figure 4. A significant mean increase over the whole population (*p* < 0.05) of recall and F1-score was provided by DEMANN, for onset and offset prediction. This improvement (*p* < 0.05) was preserved also considering signals from a single muscle, for both TA and for GL. No significant differences (*p* > 0.05) were identified in the VL signals and for all the prediction parameters.

## 4. Discussion

The present study was designed to test the capability of a novel machine-learning-based approach of estimating onset and offset timing of muscle activation. One of the main advantages of the present DEMANN approach is that the neural network was trained by means of only simulated sEMG signals (no real signal was needed to train the neural network), thus avoiding all the possible complications and costs associated with a typical experimental procedure. A further advantage was the running time. Without considering the processing time, which depends on the processing capability of the running device (in the case of the present neural network, it was less than 1 ms on an i-7 processor), once the model was trained, the maximum delay of activation prediction was 10 ms (the size of the windows). Although this paper did not explicitly target real-time applications, such a delay can be acceptable even under real-time constraints [26], making DEMANN suitable for the detection of muscle activity in sEMG-driven assistive devices, such as orthoses and exoskeletons. Otherwise, this could be an issue for the algorithmic (non-machine-learning) approaches. For example, the recent literature proposed a novel algorithm for detecting muscle activation in a time-frequency domain, based on Continuous Wavelet Transform (CWT) [11]. This study focused on quantifying the frequency content of the muscle activations and needed to detect muscle activation in the time domain in order to properly compute the frequency range (maximum and minimum). This approach could be very useful for specific aims and could open a new way to deepen the knowledge of neuromotor disorders. However, as most of the algorithm-based approaches, it was based on the computation of a threshold value in order to identify the activation onset and offset [5,6,7,8,9,10,11]. Thus, a portion of the sEMG signals must be processed to compute the threshold. This introduces a time-delay of at least the duration of the chosen portion, increasing the running time. In cyclic tasks such as walking, such a portion corresponds to a complete gait cycle. This would introduce a delay of at least 1 s, limiting the application of the approach to environments where real-time application is requested, such as in sEMG-driven exoskeletons. This is not needed in the DEMANN approach, where activations are predicted on subsequent 10 ms windows. Moreover, to identify each single gait cycle, kinematic or dynamic data are needed, such as signals from foot-switch sensors, pressure mats, stereo-photogrammetric systems, and inertial measurements units. This introduces a further complexity in experimental settings, potentially raising the costs, the time consumption, and the intrusiveness on patients. DEMANN does not suffer of these limitations, as it is based on a “blind” segmentation in short time segments.

In the present study, DEMANN proved to provide high performances in three different datasets: (1) a test bench of 864 simulated sEMG signals; (2) 103 real sEMG signals acquired in vastus lateralis during knee extension and in biceps brachii during elbow flexion; and (3) real sEMG signals from gastrocnemius lateralis, tibialis anterior, and vastus lateralis collected during 30 subjects walking. Details are reported in the following two sections.

### 4.1. Simulated sEMG Signals

DEMANN provided a high classification performance, quantified by a mean accuracy (±SD) of 97.8 ± 3.0% and supported by the accuracy = 95.3% in the worst-case scenario (SNR = 3 dB, Table 2). Differences due to increasing SNR values were very small (<4%), suggesting a good robustness to SNR variability. The classification performances of activity vs. silent area confirmed these findings (Table 3).

The effective classification capability and the efficient post-processing of model output provided mean prediction very close to 100% (Table 5). The variability of MAE in the function of α, σ, and SNR is reported in Table 6. Independently from the SNR effect, MAE increased where α and σ assumed the highest values. This means that the quality of prediction worsened, enlarging the activation time-duration, being the time support (i.e., the duration of a single activation) defined as 2 × α × σ. However, for activations lasting up to 45% of the simulated-signal duration (450 ms), MAE was <15 ms for both onset and offset predictions, except for sporadic low-SNR situations (<6 dB). MAE > 50 ms was reported mainly for those activations characterized by the concomitant conditions of time durations > 60% of the simulated-signal duration (600 ms) and SNR < 10 dB (red areas, Table 6). It is worth noticing that, in cyclic tasks such as walking, a single muscle activation longer than 50% of signal period (gait cycle, for walking) is rare. Continuous muscular recruitment longer than 60% of the gait cycle is practically not realistic during walking. Muscle groups such as ankle plantar flexors (gastrocnemius, soleus, peroneus) and knee extensors and flexors (vastii, rectus femoris, biceps femoris) are typically recruited for short periods, covering up to 35% of the gait cycle [39]. Only ankle dorsi flexors (tibialis anterior, extensor digitorum longus) may rarely present activations that last up to 50% of the gait cycle. Thus, for most practical applications, DEMANN can provide onset-offset estimation affected by MAE < 20 ms for a wide SNR range (3–30 dB), confirming a good classifier robustness for SNR variability.

The efficiency of the DEMANN approach was firstly proved versus a different machine-learning model. The support vector machine (SVM) was chosen among the models proposed in the literature as a suitable tool for this purpose [22,23,24,25]. A comparison, in the whole dataset of 864 simulated sEMG signals, specifically generated for the current experiments, showed DEMANN outperforming SVM, in terms of both onset and offset MAE (Table 4). Moreover, the DEMANN robustness was supported by comparison with the DT algorithm on the same simulated data (Table 5). DEMANN predicted offset values with better accuracy for the lowest SNR values (SNR < 6; Figure 3B). Moreover, DEMANN provided F1-score = 100% in offset prediction for SNR ≥ 10 dB; DT only for SNR ≥ 13. Likewise, mean offset MAE over the whole dataset was reduced in the DEMANN prediction, compared to DT (Table 5, *p* < 0.05). This was true also considering each single SNR value (Figure 3D); the reduction was significant (p < 0.05) for SNR = 3 and for SNR ≥ 16. An absence of statistical significance for 6 ≤ SNR ≤ 13 was likely due to the very large mean SD (28.8 ms) associated with the mean MAE computed over DT predictions in this range. Particularly relevant was the 47% reduction of MAE for SNR = 3 dB, suggesting that DEMANN improved DT performances especially in the lowest SNR values. Although an overall reduction of onset-MAE was visible in the DEMANN prediction (Figure 3C), no significant difference was detected.

One of the most reliable sEMG timing detectors reported in the literature is the wavelet-based approach described in [10]. In that study, the robustness of algorithm performances was also tested on simulated sEMG signals. However, a suitable comparison of the results of the current study with those reported in [10] was hard to accomplish because of the many differences in the generation of the simulated signals (different values of α, σ, and SNR) and in the metrics used to evaluate the algorithm performances (MAE in the present study and bias in [10]). Nevertheless, in the attempt of giving the readers further tools to evaluate the robustness of the present approach, the bias has been computed also in the present data as the relative (with sign) value of the time distance between the predicted and the ground-truth value. Results computed in the signals characterized by SNR = 20 dB (the only value in common between the present study and the one reported in [10]) were compared with those reported in [10]: mean bias was 1.7 ms for DEMANN vs. 7.1 ms in [10] for the onset and −2.8 ms for DEMANN vs. 4.1 ms in [10] for the offset. Signs “−” and “+” were adopted to indicate that the predicted event occurred earlier and later than the corresponding value in the ground-truth signal, respectively.

### 4.2. Real sEMG Signals

The dataset introduced in [3] was mainly chosen for the specific characteristics of the motor tasks (knee extension and elbow flexion), which allow for achieving a reliable detection of the onset event and consequently a trustworthy ground truth. Only onset events were tested, because the ground truth for offset events was not available in [3]. Outcomes of the application of DEMANN to this dataset are shown in Table 7. At first glance, it seems that a substantial difference exists between MAE values obtained for the simulated (Table 5) and real sEMG signals when using DEMANN. However, considering the same SNR range (3 dB ≤ SNR ≤ 12 dB), the distance between the two MAE values was strongly reduced (MAE-simulated = 19.1 ± 25.5 ms vs. MAE-real = 38.5 ± 56.4 ms); MAE and SD are about twice as many in real signals. This difference may be mainly due to a couple of reasons: (1) the neural network was trained with only simulated signals; (2) the larger variability of real sEMG signals due to the eight-shaped path followed by subjects during the experimental procedure that introduced further sEMG variability (caused by curves, reversing, deceleration, and acceleration [40]) and thus affected the performance of classification and prediction.

Table 8 highlights that the DEMANN approach globally outperformed the performance of the algorithms tested in [13], providing: (1) the lowest absolute error values over the whole 52-signal dataset (SNR ≤ 8 dB) for all considered metrics; (2) a relevant reduction of mean and median values over the whole 52-signal dataset of absolute error compared to the best value (ETKEO) reported for DT (mean 31.4%; median 21.8%), WLT (mean 28.7%; median 31.0%), CUSUM (mean 20.3%; median 31.0%), and PROLIFIC (mean 24.6%; median comparable); (3) the same result also for the signals with 6 dB < SNR < 8 dB; and 4) performances comparable with those achieved by the four algorithms, for SNR < 4 dB. As conducted in [13], this dataset was adopted to evaluate the performance of the proposed approach on sEMG signals characterized by a range of low SNR (≤12 dB). For 6 dB ≤ SNR ≤ 12 dB, absolute error was practically not affected by SNR variability (Table 7). It was reported that, in limb movement studies, time differences from stimulus to sEMG onset with neurological diseases, aging, and postural sets may be as low as 20 ms [41]. The performances of DEMANN in the SNR range from 6 dB to 12 dB complied with these requirements. For lower SNR values (<6 dB), the absolute error was proportionally increasing with decreasing SNR, up to 200 ms for SNR < 2 dB. For this SNR range, and for these specific motor tasks (knee extension and elbow flexion), all the algorithms considered in Table 8 reported high values of absolute error, not complying with the abovementioned clinical needs. However, for these very low SNR values, the identification of onset timing by visual inspection could be very hard also when performed by actual experts, as shown in [13]. Thus, onset prediction is affected not only by the reduction of algorithm performances but also by the uncertainty associated with ground truth identification. In our opinion, this consideration may contribute to explain the high values of absolute error, especially for SNR < 4 dB. This would contribute to also explain the fact that, for similar SNR (=3 dB), the mean MAE provided by DEMANN in the simulated signals was around 20 ms (Figure 3C).

Since walking is one of the most useful tasks to obtain insights on human movement, DEMANN was tested also on a dataset of sEMG data collected during 30 healthy adults walking. Despite the high data variability due to curves, reversing, deceleration, and acceleration during the eight-shaped path, prediction performances were >90% for both the onset and offset prediction (Figure 4). Performances provided by DEMANN were validated vs. the DT algorithm. Significantly higher values (*p* < 0.05, Figure 4) of recall and F1-score for onset and offset prediction showed that DEMANN outperformed the DT algorithm in correctly identifying these events. This was true (*p* < 0.05) also considering the mean values over the signals from the same muscle, in the case of TA and GL. Otherwise, for VL, no significant difference was detected between the two approaches. TA and GL are mainly ankle flexor muscles and VL is a knee extensor; it is acknowledged that ankle muscles are typically more involved in the walking task [39]. Given that differences between DEMANN and DT were significant for TA and GL but not VL, one interesting direction to follow in the future studies could be the analysis of possible muscle specificity of the present approach.

## 5. Conclusions

The present outcomes suggest the feasibility of predicting onset-offset timing of muscular recruitment of the proposed machine-learning-based method, which was able to provide high performances also in condition of large variability of the sEMG signal. The adoption of DEMANN introduced several further advantages, such as a running time compatible with real time applications, a small deterioration of event detection due to low SNR values and to a large within-signal variability of SNR, and reduced complexity of the experimental protocol associated with model training, since no real signal is needed. All these advantages make this approach suitable for clinical practice and for being included in the procedure for controlling sEMG-driven assistive devices, such as orthoses and exoskeletons.

The DEMANN approach was validated in simulated sEMG signals and in real sEMG signals acquired in young able-bodied subjects, but not in elderly and pathological populations. This is acknowledged as a limitation of the present study. Future studies will be focused on assessing the reliability of the DEMANN approach to provide a robust prediction of activation events also in these populations and on the possible improvements to implement for adapting the model to different conditions and environments. While the present study showed that relatively simple supervised methods, such as shallow neural networks, can be suitable for muscle activation detection, further experiments should be made to determine an optimal classifier to embed in the detecting system.

## Figures and Tables

**Table 1:** Mean classification accuracy in the simulated test dataset associated with different inputs.

Input	F1-Score ± SD (%)			
	Activity Area	Silent Area	Macro	Weighted
LE	95.0 ± 0.4	87.9 ± 0.8	91.4 ± 0.6	92.8 ± 0.5
RMS	96.4 ± 0.3	91.5 ± 0.6	93.9 ± 0.4	94.9 ± 0.4
CWT	98.0 ± 0.2	95.5 ± 0.4	96.8 ± 0.3	97.3 ± 0.2
LE + RMS + CWT	98.3 ± 0.1	96.0 ± 0.3	97.2 ± 0.2	97.6 ± 0.2

**Table 2:** Mean classification accuracy stratified for different SNR.

SNR (dB)	Accuracy (%)
3	95.3 ± 4.8
6	96.2 ± 4.3
10	97.3 ± 3.3
13	98.1 ± 2.1
16	98.4 ± 2.0
20	98.9 ± 1.4
23	99.2 ± 0.9
26	99.1 ± 1.0
30	99.1 ± 0.7
Mean ± SD	97.8 ± 3.0

**Table 3:** Mean classification performances computed in the test set separately for the activity area and the silent area, stratified for different SNR.

**Activity Area**			
**SNR (dB)**	**Precision (%)**	**Recall (%)**	**F1-Score (%)**
3	95.1 ± 7.6	91.6 ± 10.3	92.7 ± 6.1
6	96.0 ± 6.1	93.4 ± 8.2	94.2 ± 4.2
10	97.8 ± 3.7	94.2 ± 7.3	95.7 ± 3.8
13	98.8 ± 2.3	95.2 ± 6.2	96.7 ± 3.2
16	98.8 ± 2.0	96.5 ± 4.5	97.5 ± 2.4
20	98.7 ± 1.5	97.9 ± 3.1	98.2 ± 1.5
23	98.9 ± 1.6	98.3 ± 2.4	98.5 ± 1.3
26	98.5 ± 2.2	98.5 ± 2.1	98.5 ± 1.4
30	98.4 ± 2.0	98.3 ± 4.2	98.3 ± 2.4
Mean (±SD)	97.9 ± 4.0	96.0 ± 6.4	96.7 ± 3.8
**Silent Area**			
**SNR (dB)**	**Precision (%)**	**Recall (%)**	**F1-Score (%)**
3	94.8 ± 8.0	97.8 ± 4.7	96.0 ± 5.0
6	94.8 ± 9.5	98.8 ± 1.8	96.5 ± 5.4
10	96.3 ± 6.5	99.3 ± 1.3	97.6 ± 3.7
13	97.4 ± 3.8	99.4 ± 1.1	98.4± 1.9
16	97.7 ± 4.6	99.5 ± 0.9	98.6 ± 2.6
20	98.4 ± 3.8	99.5 ± 0.7	98.9 ± 2.0
23	99.1 ± 2.1	99.6 ± 0.7	99.3 ± 1.1
26	99.0 ± 2.8	99.3 ± 1.0	99.1 ± 1.5
30	99.3 ± 1.4	99.3 ± 0.9	99.3 ± 0.7
Mean (±SD)	97.4 ± 5.6	99.2 ± 1.9	98.2 ± 3.3

**Table 4:** Mean (±SD) performances of the onset and offset prediction provided by DEMANN and SVM over all the simulated sEMG signals.

	DEMANN		SVM	
	Onset	Offset	Onset	Offset
MAE (ms)	10.0 ± 17.5 *	10.1 ± 17.3 ^§^	20.6 ± 28.2 *	19.3 ± 23.8 ^§^
Precision (%)	99.0 ± 9.6	99.4 ± 7.4	97.0 ± 16.9	98.5 ± 11.9
Recall (%)	99.2 ± 9.0	99.5 ± 6.8	97.1 ± 16.8	98.6 ± 11.7
F1-score (%)	99.0 ± 9.2	99.4 ± 9.6	97.0 ± 16.8	98.5 ± 11.8

* means that the difference between the two mean onset values is statistically significant (*p* < 0.05); ^§^ means that the difference between the two mean offset values is statistically significant (*p* < 0.05).

**Table 5:** Mean (±SD) performances of onset and offset prediction provided by DEMANN and DT over all the simulated signals.

	DEMANN		DT	
	Onset	Offset	Onset	Offset
MAE (ms)	10.0 ± 17.5	10.1 ± 17.3 *	11.5 ± 21.9	16.1 ± 26.9 *
Precision (%)	99.0 ± 9.6	99.4 ± 7.4	98.5 ± 12.1	96.9 ± 17.4
Recall (%)	99.2 ± 9.0	99.5 ± 6.8	98.4 ± 12.3	96.8 ± 17.4
F1-score (%)	99.0 ± 9.2	99.4 ± 9.6	98.5 ± 12.2	96.9 ± 17.4

* means that the difference between the two mean values is statistically significant (*p* < 0.05).

**Table 6:** Variability of MAE in the function of simulated-signal parameters α, σ, and SNR (dB) for onset and offset prediction.

**Onset—MAE (ms)**												
	**σ = 50 ms**				**σ = 100 ms**				**σ = 150 ms**			
**SNR**	**α = 1**	**α = 1.5**	**α = 2**	**α = 2.4**	**α = 1**	**α = 1.5**	**α = 2**	**α = 2.4**	**α = 1**	**α = 1.5**	**α = 2**	**α = 2.4**
3	7.2	6.9	8.9	21.0	11.4	15.1	18.0	60.8	21.7	10.3	37.3	72.8
6	8.4	11.1	13.5	18.2	6.2	9.6	12.6	52.6	5.2	5.6	33.9	87.4
10	5.1	6.1	7.1	10.0	5.9	12.1	9.4	42.6	4.6	1.6	32.5	62.4
13	3.1	2.7	3.0	11.1	3.3	7.1	8.1	31.6	8.2	4.4	11.8	35.0
16	1.8	2.5	6.2	9.5	2.0	3.9	9.9	28.5	2.0	8.4	4.6	33.1
20	1.2	1.9	4.0	5.4	1.1	3.1	4.7	13.8	2.3	3.8	4.6	20.3
23	1.8	2.8	4.6	4.9	1.3	1.7	3.8	8.7	1.2	3.4	4.5	3.9
26	2.6	1.8	3.3	6.4	2.4	2.3	5.1	7.0	2.0	4.3	2.9	12.0
30	1.1	2.0	5.7	9.8	2.0	3.4	3.8	3.0	2.2	4.8	5.0	11.5
**Offset—MAE (ms)**												
	**σ = 50 ms**				**σ = 100 ms**				**σ = 150 ms**			
**SNR**	**α = 1**	**α = 1.5**	**α = 2**	**α = 2.4**	**α = 1**	**α = 1.5**	**α = 2**	**α = 2.4**	**α = 1**	**α = 1.5**	**α = 2**	**α = 2.4**
3	5.2	6.6	12.1	29.1	9.8	14.8	34.8	55.9	10.2	13.3	35.1	102.3
6	3.3	10.1	14.6	15.5	8.2	3.0	22.9	45.4	5.8	8.8	36.4	75.7
10	1.5	2.9	6.4	19.6	2.5	5.2	8.5	44.9	6.3	5.0	36.8	66.6
13	4.4	7.4	3.8	17.2	3.4	2.5	10.0	49.3	1.0	5.8	12.3	36.0
16	1.2	9.3	7.4	10.5	2.6	3.6	3.6	23.4	2.7	6.8	4.4	37.5
20	1.6	4.4	4.6	6.8	3.3	3.0	3.9	11.3	1.4	4.6	12.3	31.9
23	2.2	3.6	5.3	6.0	1.3	2.5	5.1	12.7	1.5	2.5	2.7	26.5
26	2.8	3.1	5.3	4.6	1.6	5.2	2.6	4.8	2.6	4.6	6.1	24.5
30	2.0	9.5	1.8	7.7	2.1	3.9	4.9	6.6	1.6	2.4	6.3	6.2

All the areas with different levels of green indicate MAE values < 10 ms. Progressively darker green indicate progressively lower MAE. All the yellow, orange, and red areas indicate MAE values ≥ 10 ms. Progressively darker colors indicate progressively higher MAE. The value of 10 ms was chosen since it was the mean MAE value over the whole dataset (Table 5).

**Table 7:** Absolute error of onset prediction in the function of SNR ranges in terms of mean, standard deviation (SD), median, 25-percentile, and 75-percentile.

SNR (dB)	Number of Signals	Mean (ms)	SD (ms)	Median (ms)	25-Perc (ms)	75-Perc (ms)
≤2	6	209.9	182.0	131.6	66.9	368.8
2 ÷ 4	10	187.5	163.7	116.0	60.4	338.0
4 ÷ 6	15	76.7	53.7	77.6	32.7	107.4
6 ÷ 8	21	24.0	27.7	13.2	6.8	32.2
8 ÷ 10	20	15.8	16.9	11.5	3.9	16.4
10 ÷ 12	6	12.2	2.9	12.9	11.6	14.3
≤8	52	92.1	120.3	54.2	13.2	93.9
>8	26	14.9	14.6	12.0	7.1	14.6

**Table 8:** Comparison among the absolute errors of the onset prediction provided in the same population by DEMANN approach and by the four algorithms introduced in Section 3.2. The best values for each parameter and each SNR are highlighted in bold.

SNR (dB)	DEMANN	DT		WLT		CUSUM		PROLIFIC	
		TKEO	ETKEO	TKEO	ETKEO	TKEO	ETKEO	TKEO	ETKEO
**Mean (ms)**									
≤2	209.9	733.5	243.7	504.5	139.8	827.1	**126.6**	357.9	303.4
2 ÷ 4	187.5	225.5	154.0	191.5	**145.9**	1143.8	222.8	460.0	185.5
4 ÷ 6	**76.7**	201.3	101.3	248.5	165.2	708.1	93.8	371.4	123.4
6 ÷ 8	**24.0**	182.3	116.9	158.8	92.2	618.0	65.5	229.7	39.4
≤8	**92.1**	259.7	134.2	230.9	129.1	769.2	115.1	410.4	122.2
**SD (ms)**									
≤2	182.0	456.4	381.0	578.5	**66.9**	584.3	91.9	534.6	519.4
2 ÷ 4	163.7	115.0	**92.0**	254.5	146.3	489.2	170.4	392.2	185.5
4 ÷ 6	**53.7**	266.9	106.2	335.4	272.5	492.2	92.0	453.3	123.4
6 ÷ 8	**27.7**	305.3	229.8	311.4	170.2	579.2	54.8	462.0	39.4
≤8	120.3	330.1	203.6	352.9	192.3	558.7	**114.8**	443.5	122.2
**Median (ms)**									
≤2	131.6	765.4	**92.5**	208.3	133.3	999.0	149.7	111.1	104.0
2 ÷ 4	116.0	231.2	125.0	121.3	**95**	1134.5	148.7	396.7	136.0
4 ÷ 6	77.6	104.0	58.6	122.6	104.9	793.5	55.7	93.8	**50.8**
6 ÷ 8	**13.2**	69.8	41.5	61.0	35.2	729.0	48.8	135.7	36.6
≤8	**54.2**	109.6	69.3	116.9	78.6	958.0	78.6	137.9	54.9
**25-Percentile (ms)**									
≤2	66.9	400.9	42.9	127.4	126.5	153.8	90.8	**57.6**	56.6
2 ÷ 4	60.4	121.1	87.9	**30.3**	33.7	883.3	128.9	140.1	124.5
4 ÷ 6	32.7	47.8	27.6	71.2	31.7	257.1	**23.4**	40.4	20.7
6 ÷ 8	**6.8**	28.3	25	19.4	7.8	35.9	32.3	30.5	11.7
≤8	**13.2**	45.2	31.5	33.0	25.2	100.3	40.3	46.6	24.9
**75-Percentile (ms)**									
≤2	368.8	1042.9	**192.2**	861.3	197.7	1182.1	239.7	409.2	157.7
2 ÷ 4	338.0	309.1	245.6	191.9	**188.5**	1483.9	298.3	746.1	173.3
4 ÷ 6	107.4	231.9	146.6	223.4	128.5	1124.8	131.2	680.9	**88.6**
6 ÷ 8	**32.2**	107.5	94.2	122.4	130.5	1115.2	80.9	884.7	54.8
≤8	**93.9**	304.7	146.5	192.9	146.5	1181.4	152.6	756.6	125.5

## Data Availability

Data supporting reported results can be found by contacting the corresponding author.
